# Development of the Chilean front-of-package food warning label

**DOI:** 10.1186/s12889-019-7118-1

**Published:** 2019-07-08

**Authors:** Marcela Reyes, María Luisa Garmendia, Sonia Olivares, Claudio Aqueveque, Isabel Zacarías, Camila Corvalán

**Affiliations:** 1 Institute of Nutrition and Food Technology (INTA), University of Chile, Av. El Líbano 5524, Macul, 138-11 Santiago, Casilla Chile; 2 Business School, Universidad Adolfo Ibáñez, Santiago, Chile

**Keywords:** Front-of-package, FoP, Warning label, Stop signs, Chilean labelling law

## Abstract

**Background:**

Front-of package (FoP) nutrition labels are an option to guide consumer’s decision at the point of food purchase. Chile was the first country worldwide to implement a FoP warning label and thereafter several countries have followed this model. The objective of this study is to describe the process of development of the Chilean FoP warning label.

**Methods:**

A stepwise study was conducted including literature review, qualitative phase (lay audience & expert group meetings) and quantitative phase in women/adolescents from low-middle-socioeconomic status neighborhoods, Santiago, Chile (2 sub-studies, using point-of-sale questionnaires). Outcomes were prototype visualization, ease of understanding, and ability to modify purchase behavior.

**Results:**

The literature review and qualitative phase provided information on general text (e.g. short wording) and design characteristics (e.g. use of a logo, use of red or black colors); based on these characteristics 15 prototypes were created and quantitatively tested. In the first survey, a black-&-white stop sign and a black-&-white hand were preselected; in the second survey, the stop sign stating ‘Excess of <*nutrient*>’ had significantly better performance than the hand in terms of visualization, intention to purchase, and ability to modify intended purchase. Due to legal reasons the “excess of” was replaced by “high-in” in the final implementation of the law.

**Conclusions:**

A simple black-&-white stop sign warning label was the best option to flag pre-packaged foods with an excess of energy or nutrients of concern for non-communicable diseases; this FoP warning label was implemented in Chile in June 2016 as part of the Chilean Food Labeling and Marketing Law.

**Electronic supplementary material:**

The online version of this article (10.1186/s12889-019-7118-1) contains supplementary material, which is available to authorized users.

## Background

Currently, Chile is considered a post-transitional country because obesity and diet-related non-communicable diseases (NCDs) are the major causes of death and disability while stunting, and anemia have been almost eradicated [1]. According to the last national health survey (2017), almost 3/4 of Chilean adults are either obese or overweight, with a greater prevalence among low socioeconomic status (SES) people [2]. Moreover, over 50% of 6-7-year-old children are either obese or overweight [1]. These figures illustrate the NCDs epidemic the country is undergoing, which has been also reported for other in other developing economies [3].

During the last two decades Chile has implemented several actions to halt the obesity epidemic and improve Chileans’ diet; however, most of them rely on individual responsibility and thus, obesity has continued to increase relentlessly. To move into more structural regulations to ensure healthier food environments, in 2012 the National Congress of Chile approved the Food Labelling and Marketing Law. The Law states that pre-packaged foods with high content of energy and nutrients of concern for NCDs (i.e. sugars, saturated fats, or sodium) must have a front-of-package (FoP) warning label that inform the consumer of this condition; also, these foods cannot be promoted to children under 14 years and cannot be sold at schools/nurseries or provided as part of school/nurseries food programs [4–6].

FoP labelling has been defined as strategy to improve consumer’s information at the point of purchase and thus, it is one of the suggested regulatory actions to promote healthier diets. Recent initiatives have been based on highlighting positive food attributes from a health perspective [7] such as the Guideline Daily Amounts (GDA) [8] and health-endorsed logos (i.e. Nordic Keyhole) [9], or a combination of positive and negative attributes such as the traffic-light experience [10]; all these systems had shown an increase in the use and understanding of nutrition labelling compared to traditional nutrient fact panels [11]. In the case of Chile, initially the United Kingdom (UK) traffic-light was proposed as the FoP labelling system. This proposal was in line with the international experience but focus groups conducted during the preliminary discussions of the law showed the system was difficult to understand by Chileans [12] who tended to average the different colors; several other studies have now confirmed these findings [13–15]. Moreover, food industry also confronted the use of the traffic-light in favor of GDA; therefore, a more general description of the label was agreed for the approval of the Law in the senate, with no further specification on the design or wording, but with the indication the label should clearly flag packaged foods with high content of key nutrients and warn about that condition [4]. At the time of discussion of the Chilean Law no previous experience on the use of an exclusive warning FoP label on packaged foods existed globally and therefore, there was a need of designing a new alternative.

This article describes the process of development of the warning label that was finally implemented as part of the regulation, which started in June 2016. A multi-method approach was used to test visualization, understanding, and ability to modify intended purchase of different prototypes of warning labels. The study only addresses the graphical design of a warning label and does not provide a comparison with other types of FoP labelling schemes as the goal was to provide a concrete proposal of warning label to implement the Chilean Law. Since the implementation of the Chilean FoP warning label, several other countries have implemented similar FoP models or are in the process of discussion of their implementations [16–20]; therefore, this article is relevant for several regulatory discussions taking place worldwide.

## Methods

### Study overview

This was a stepwise study aiming to develop a FoP warning label for pre-packaged foods which was easy to visualize, understandable, and able to modify intended purchase. The study was performed in subsequent phases: (i) *Literature Review*, that provided basic information on design aspects of the label, (ii) *Qualitative Phase* (*Lay Audience* and *Expert Group Meetings*), that captured people’s perception on design aspects of the label that helped for narrowing down the options to be tested in the (iii) *Quantitative Phase (Sub-studies #1 and #2)*, that provided information of the performance of the different prototypes in terms of visualization, understanding, and purchase behavior. Research was conducted in collaboration with a design company with experience on designing food packages. The study was done between August and December 2012. A research team composed by scholars from public health, epidemiology, nutrition and food labelling, marketing, and social marketing, participated in the design, data collection, data analysis and data interpretation. A Spanish report is available upon request [21].

#### Literature review

A Literature Review was conducted to identify impact, effectiveness-related factors, and real experiences of warning labels in foods/beverages. Given the scarce information available, the search was expanded to include tobacco and alcohol since there are experiences using warning messages among those products as public health measures. Scientific literature from marketing, health and nutrition areas were examined using search engines like Pubmed and ISI Web of Knowledge. Keywords included: ‘Salt AND Warning messages’, ‘Salt AND Advertisement message’, ‘Avoid message AND Sugars’, ‘Consumer awareness campaign AND Fats’, ‘Tobacco and Warning messages AND Evaluation of impact’. References of articles and websites were also reviewed as well as articles that cited those references. Websites of international institutions related to health and nutrition were reviewed (eg. American Heart Association, World Health Organization, National Institute for Food and Nutrition Science, World Action on Salt and Health). The review stopped when saturation was achieved.

#### Qualitative phase

##### Lay audience group meetings

After the literature review, we performed six focus group meetings (60-90 min each) using the Metaplan technique [22], each set of participants took part in a unique meeting. Briefly, the Metaplan technique is a method for facilitating group discussion that allows organizing and providing structure of ideas and opinions obtained from a brainstorming process; categorization and ranking of ideas may allow that new connections emerge. All meetings were performed at the Institute of Nutrition and Food Technology (INTA), conducted by two of the authors (S.O & I.Z.) with two research assistants (C.G.G. and E.V. both with M.Sc.); the researchers have extensive experience performing Metaplan technique in nutrition-related areas. The objective of these meetings was to define text and design characteristics to be included in the prototypes to be tested in the “*Expert Group Meeting*”; a secondary objective was to choose a food that consumers considered neutral (not considered a priori healthy or unhealthy) to test the prototypes in the *Quantitative Phase*. Group Meetings were conducted among 18-59y women from low-middle SES, who were responsible for food purchase at home (*n* = 3 groups, 5-8 people per meeting), and 12-17y adolescents from low-middle SES (*n* = 2 groups, 8 people per meeting) to consider opinions of target groups of the Labelling and Marketing Law. Participants were recruited from low-middle income neighborhoods using recruitment posters inviting to participate in a study to learn about the barriers for eating healthy; a community member was hired to verify eligibility criteria of interested participants (including no previous relationship with the researchers in charge of the study). Sessions were conducted following the Metaplan technique. Briefly, facilitators started each session with a short introduction of the objectives of the session. Participants were then invited to provide their opinions regarding text and design options for the labels writing their ideas on color-cards; each color represent different aspects to be considered (such as figure, colors, wording, etc. of the label). Facilitators were then in charge of organizing the cards of on a panel and guiding the group to prioritize answers on each of the topics. Research assistants registered conclusions and sessions were also audio recorded as a back-up. Researchers analyzed the conclusions in each of the sessions and together with a food design company developed 24 prototypes that fulfilled the recommendations obtained from the Metaplan; these 24 prototypes were tested on the next phase (see Additional file 1).

##### Expert group meeting

We invited more than 50 experts from different areas, public and private sectors, and universities to participate in an Expert Group Meeting to refine the selection of the prototypes to be tested in the Quantitative Phase (targeted invitations were done via email or phone). The final group was composed by 14 experts of nutrition, social science, public policy, marketing and trade scholars, as well as representatives of consumer’s organization, scientific societies, government and senate; most of them have no prior formal connection with the researchers of this study. The 2-h meeting was held at INTA and conducted by 3 of the authors (CC, MR, MLG) following a predefined structure of items to be addressed. Experts were debriefed on the final goal of the study and then presented with the 24 prototypes defined in the *Lay Audience Group Meetings* (see Additional file 1). For each of the prototypes, experts were asked to score color, shape, text, easy of visualization, potential impact on purchase behavior, and feasibility to be implemented. The meeting was recorded, and field notes were taken during and after the meetings. Researchers analyzed the scores given by the experts and complemented this information with notes of the discussions and specific suggestions. Based on the suggestions, researchers together with the food design company created 15 prototypes of warning labels to be tested in the *Quantitative Phase*.

#### Quantitative phase

##### Sub-study #1

A first Sub-study was conducted in 600 women to select two prototypes (out of 15) to be further tested in the subsequent phase (Sub-study #2). Each of the 15 prototypes were displayed in a yogurt (identified as a neutral product in the *Lay Audience Meeting*) with a fictitious label and brand (see Additional file 2). Each prototype was shown to 40 women, recruited at the entrance of 30 supermarkets from low-middle SES neighborhoods of Santiago, Chile. Using a 10-min survey (see Additional file 3), participants provided information on visualization, understanding, and purchase intention. Sociodemographic information, self-reported weight and height, and the presence of cardiometabolic conditions in the family were also asked. Inclusion criteria for the study were: women 18-59y, living in the neighborhoods where the interview was done, being responsible for food purchases, and purchasing > 3 units of yogurts/week. There were no exclusion criteria. Sample size (*n* = 40) allowed detecting 15% difference in means, with a standard deviation of 25% (alpha error = 5%, beta error = 20%).

##### Sub-study #2

A second sub-study was conducted in 700 women to identify which of the two prototypes selected in the Sub-study #1 behaved better (same outcome variables, 350 women per prototype). We also tested how to display warning label(s) on foods with excessive content in several nutrients (e.g. high in energy + high in sugars) (see Additional file 4). Methodology, and inclusion criteria were the same of Sub-study #1. In order to assess performance of the labels in vulnerable groups, we also interviewed 300 12-17y adolescents and 300 low SES adult women (recruited at the entrance of 12 supermarkets from low SES neighborhoods of Santiago, Chile).

##### Outcome variables

Four outcome variables were considered for the Quantitative Phase: i) Visualization, ii) Understanding, iii) Intended purchase (Likert-like scale), iv) Ability to modify intended purchase (numerical variable). Additionally, we tested which was the best way of informing when a food had multiple nutrients in excess by comparing the i) Visualization, ii) Understanding and iii) Intention to purchase, of a prototype with 3 labels and a prototype with one label (Additional file 4). Questions used for estimating each of these scores and the potential range of values of the scores are presented in Table 1.

**Table 1:** Description and calculation of visibility, understanding, intended purchase, ability to modify intended purchases and nutritional scores and other sociodemographic variables used to the evaluate performance of labels in Chile

Scores	Calculation	Questions	Score range
Visibility	Percentage of participants who spontaneously identified the warning label over the total number of participants.	Q9. Is there anything on the label of this product that attracts your attention? (three more important)	0-100%
		The interviewed identified the warning label?	
		Yes/No	
Understanding	Addition of individual values of Q11, Q12, Q13 & Q14. For Q13 assigned values were either 0 (options 1-4) or 5 (option 5).	Q11. How easy to understand is this message?	3-20
		1. Very difficult to understand = 1	
		2. Difficult to understand = 2	
		3. Indifferent = 3	
		4. Easy to understand = 4	
		5. Very easy to understand = 5	
		Q12. According to this message, the excessive consumption of this product is (…)	
		1. Very healthy = 1	
		2. Healthy = 2	
		3. Indifferent = 3	
		4. Unhealthy = 4	
		5. Very unhealthy = 5	
		Q13. According to this message ¿which of the following nutrients is excessive? Score 0 or 51. Calories = 0	
		2. Sodium = 0	
		3. Calcium = 0	
		4. Saturated fats = 0	
		5. Sugar = 1	
		Q14. According to this message, you should consume (…)	
		1. A lot more of this product = 1	
		2. A bit more of this product = 2	
		3. Same as usual = 3	
		4. A bit less of this product = 4	
		5. Nothing at all of this product = 5	
Intended purchase score	Mean (SD) of the values obtained in Q16 (values range: 1 to 5).	Q16. If the yogurt you usually buy had this message, would you buy it?	1-5
		1. I would buy it for sure	
		2. It is likely that I would buy it	
		3. Indifferent	
		4. It is unlikely that I would buy it	
		5. I would not buy it	
Ability to modify intended purchase	1 – (Q17 / Q5) × 100 (weekly for women, monthly for adolescents).	Q17. If the yogurt you usually buy had this message, how many products would you buy per week? ________ units	0-100%
		Q5. How many units of yogurt do you buy per week? _______ units	
Nutritional conscience	Addition of individual values from Q22 and Q23.	Q22. When you buy foods, how important are the nutritional characteristics of the product in your purchase decision?	2-10
		1. Not important at all	
		2. Not very important	
		3. Indifferent	
		4. Important	
		5. Very important	
		Q23. When you buy packaged foods, how often you read the nutrition fact panel	
		1. Never	
		2. Almost never	
		3. Sometimes	
		4. Frequently	
		5. Always	
Education level	Percentage of women who answered options 5, 6, 7, 8 or 9 in Q8.	Q8. Are you studying currently?	0-100%
		1. Yes	
		2. No	
		If question 8 = Yes→ What’s your current educative level?	
		If question 8 = No→ What’s your last approved level?	
		1. Never assisted	
		2. Special education (differential)	
		3. Primary of preparatory (old system)	
		4. Primary school	
		5. Humanity or Commercial technique, Industrial or Normalist (old system)	
		6. Secondary school	
		7. Superior level technic	
		8. Professional	
		9. Master or PhD	
		99. Do not know	
Body mass index	Q25 / (Q24 x Q24); the mean value of the range was used if a specific number was not provided.	Q25. Which is your weight?_________ kilograms	
		Alternatively, a weight range can be selected	
		1. < 45 kg	
		2. 46 - 60 kg	
		3. 61 - 70 kg	
		4. > 70 kg	
		Q24. Which is your height?_________ meters	
		Alternatively, a height range can be selected	
		1. < 1.40 m	
		2. 1.41 - 1.50 m	
		3. 1.51 - 1.60 m	
		4. > 1.61 m	
Comparative prototype score for multiple critical nutrients in excess	Addition of individual values of Q28a, 28b and 28c. When a given prototype was mentioned in first place the assigned value was = 1, when a given prototype was mentioned in second place the assigned value was = 0, when both prototyped were mentioned in first place the assigned value for each one was = 1.	Q28a. In the case that more than one nutrient is excessive, which of these 2 ways of presenting the message is easier for you to see?	0-3
		1. Option 1	
		2. Option 2	
		Q28b. In the case that more than one nutrient is excessive, which of these 2 ways of presenting the message is easier for you to understand?	
		1. Option 1	
		2. Option 2	
		Q28c. In the case that more than one nutrient is excessive, which of these 2 ways of presenting the message would have greater influence in your purchase decision?	
		1. Option 1	
		2. Option 2	

### Data analyses

Qualitative Phase: records from the Lay Audience Groups and Expert Group Meetings’ were used for content analysis. Specially trained research assistants coded answers to specific questions that addressed different design aspects of the FOP, created an Excel sheet (Microsoft Corporation, Redmond, WA, USA) and tabulated the frequency of each of the answers. Most frequent answers were considered for the design of the prototypes tested on the next phase. Quantitative Phase: outcome variables were calculated as described in Table 1. In the Quantitative Sub-study #1, chi-square was used to compare easy of visualization (%) and ANOVA & Bonferroni to compare means in understanding score, intended purchase score and ability to modify intended purchase among the 15 prototypes. In the Quantitative Sub-study #2 chi-square was used to compare visualization (%) and T-test was used to compare means in understanding score, intended purchase score and ability to modify intended purchase between the 2 prototypes. A *p*-value < 0.05 was considered statistically significant. Analyses were performed using SPSS 20.0 and STATA 11.2.

### Ethics

The Institutional Review Board from INTA, University of Chile, approved all phases of the study. Informed consent was signed by experts, women and legal guardians of the adolescents; minors signed an assent for participation. A small gift was given to participants of the group meetings as compensation.

## Results

### Literature review

At the time of the literature review the international experience using warning labels on packaged foods was very scarce. We could identify one experience in Finland, in which specific food items (i.e. butter, margarine, hot dog, bread, and cereal) with high content of sodium had to include a warning message ‘high in salt’ [23]. In UK the traffic-light system (green, yellow and red colors depending on the amount of specific nutrients) had been optionally used since 2007 [24]; however, that system is not properly a warning label because it includes a positive message (i.e. green color). The literature review indicated that warning messages were aimed to reduce the quantity and magnitude of exposure to risk factors and therefore, should be able of: (a) attracting people’s attention, and (b) providing easy-to-understand information to improve people’s decisions. Several design and text characteristics of the warning labels were recognized as improving the label’s performance. The presence of a logo [25, 26], a size > 25% of the FoP surface [25, 26], a location in the right upper corner, including a perimeter to clearly differentiate it from the rest of the package elements [26], and using contrasting colors and thick letters [12, 26, 27] were associated to greater success in visualization and use of the label. Regarding text, the use of a signal word (i.e. warning, caution, advice, danger), an ‘identification of the risk’ (i.e. high content of …), an ‘explanation of the consequences’ (i.e. it can cause diabetes), and ‘recommendations to avoid risks’ (i.e. moderate the intake) [28, 29] were considered important for achieving their purpose. Other important aspects were the use of simple language [30–32] and a layout [33, 34], using a negative framing (i.e. excessive consumption can cause hypertension) [25, 26, 35], periodically updating of the message [26, 36, 37], and inclusion of a reputable and non-authoritarian certifying source [12, 32, 38, 39]. Finally, a communication campaign was also highly recommended [12, 27, 33].

### Qualitative phase

#### Lay audience group meetings

All participants expressed interest in having warning labels on packaged foods and having easy-to-interpret information at the point-of-purchase. Signal words like “peligro (danger)” and “advertencia (warning)” were preferred by most mothers and adolescents; however, some participants found “danger” to be too strong to be used in foods, and others found that “warning” could be linked to the tobacco control campaigns. "Alto (High)" was also suggested, but some adolescents declared that its use wouldn’t change their purchase intention and some mothers associated it to a positive message (i.e. high in vitamins). Most participants preferred an octagon or triangle as the label shape. Red, orange and yellow were the preferred colors, but some participants preferred a black label given it could be more easily distinguished in packages of all colors. Most participants stated that FoP should be certified by a regulatory governmental agency to ensure credibility, being the Ministry of Health (MoH) the preferred one. Finally, participants wanted warning labels informing on causes, consequences, and even potential actions to revert the exposure to nutrients of concern. As result of these meetings, and considering the literature review, 24 prototypes were designed to be further tested with the expert group meeting (see Additional file 1).

#### Expert group meeting

Experts preferred “Exceso (Excess of)” over “High in” given that the latter was already used as a positive health claim. Overall, experts suggested to keep the wording of labels as short as possible. There was no agreement whether the wording should be playful or strong, nor whether children/adolescent or mothers should be the target public of the warning labels and campaigns. Regarding the design, most experts liked the idea to include a logo. Red was the favorite color because of its association to traffic-lights, but black was also suggested given the contrast with most food packages. Experts ranked warning label prototypes, but they mainly suggested new and simpler ideas. A hand and a stop sign were preferred as shapes. As result of the expert group meeting, and considering what was learned in previous phases, 15 prototypes were designed to be tested in the *Quantitative Phase*. Prototypes included different combinations of shape (hand vs stop sign), color (red-&-yellow vs black-&-white), ‘signal word’ (exclamation mark vs warning vs "atención (attention)" vs none), ‘explanation of consequences’ (yes vs no), size (10 vs 20% of FoP surface), and coexistence with health claims (see Table 2).

**Table 2:**
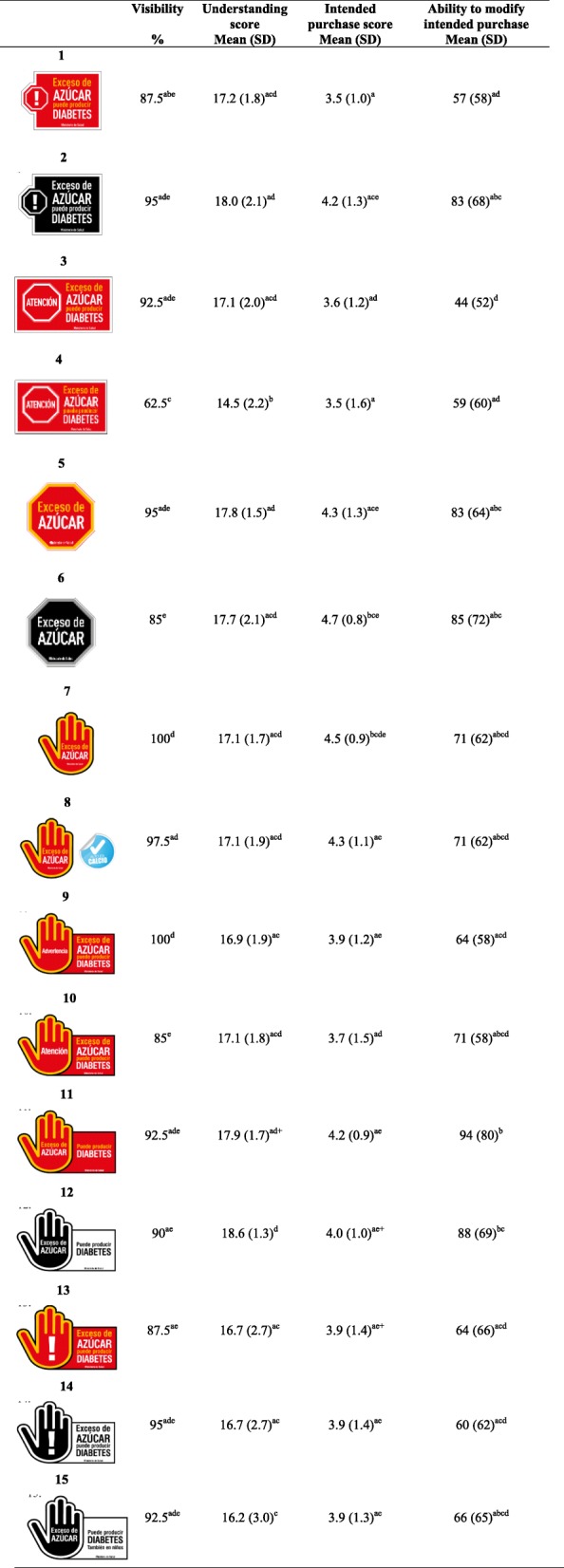
Visibility, understanding, intended purchase, ability to modify intended purchases scores of 15 warning label prototypes in Chile (sub-study #1; *n* = 600 women)

Text of prototypes (1), (2), (11), (12), (13) & (14) reads: Exceso de azúcar. Este producto puede producir diabetes. Ministerio de Salud = Excess of sugar. This product may cause diabetes. Ministry of health;

Text of prototypes (3), (4), & (10) reads: Atención. Exceso de azúcar. Este producto puede producir diabetes. Ministerio de Salud = Attention. Excess of sugar. This product may cause diabetes. Ministry of health;

Text of prototypes (5), (6), (7) & (8) reads: Exceso de azúcar. Ministerio de Salud = Excess of sugar. Ministry of health. Prototype (8) also includes a positive seal stating: Alto en calcio = High in calcium;

Text of prototype (9) reads: Advertencia. Exceso de azúcar. Este producto puede producir diabetes. Ministerio de Salud = Warning. Excess of sugar. This product may cause diabetes. Ministry of health;

Text of prototype (15) reads: Exceso de azúcar. Este producto puede producir diabetes, también en niños. Ministerio de Salud = Excess of sugar. This product may cause diabetes, also in children. Ministry of health

Chi-square was used to compare visibility (%) and ANOVA & Bonferroni to compare means in understanding score, intended purchase score and ability to modify intended purchase among the 15 prototypes

Same letters correspond to *p*-value > 0.05

Visibility, Understanding score, Intended purchase score, and Ability to modify intended purchase: greater values represent better performance

### Quantitative phase

#### Sub-study #1

Five prototypes showed greater visualization, Understanding, Intended purchase score, and Ability to modify intended purchase (i.e. prototypes 2, 5, 6, 11, and 12; see Table 2). Based on these results and on the overall better results for black-&-white prototypes, compared to red-&-yellow prototypes (data not shown), two prototypes were selected for further testing: the stop sign and the hand explaining health consequences (both with a black-&-white color combination).

#### Sub-study #2

Table 3 shows the results of the comparison between the two prototypes selected. The stop sign had significantly better results than the hand in every outcome. Results from low SES women and adolescents were similar (data not shown), with the exemption of Ability to modify intended purchase, which was greater for the hand than for the stop sign among adolescents (67% vs 47%, respectively). When testing the best way to display > 1 nutrient in excess (see Additional file 4), the option with one label per nutrient (i.e. three labels total) showed four times greater performance in the Comparative prototype score than the alternative using only one label for several nutrients: 2.4 (1.0) vs 0.6 (1.0), respectively.

**Table 3:** Visibility, understanding, intended purchase, ability to modify intended purchases scores of 2 warning label prototypes in Chile (Sub-study #2; *n* = 700 women)

	Visibility %	Understanding score	Intended purchase	Ability to modify intended purchase
		Mean (SD)	Mean (SD)	Mean (SD)
1	82.9	17.6 (2.6)	4.2 (1.3)	77.0 (58.0)
2	80.3	16.8 (3.0)	3.9 (1.3)	69.8 (53.0)
*p*-value	< 0.001	< 0.001	0.006	0.030

Text of prototype (1) reads: Exceso de azúcar. Ministerio de Salud = Excess of sugar. Ministry of health

Text of prototype (2) reads: Exceso de azúcar. Este producto puede producir diabetes. Ministerio de Salud = Excess of sugar. This product may cause diabetes. Ministry of health

Chi-square was used to compare visualization (%) and T-test was used to compare means in understanding score, intended purchase score and ability to modify intended purchase between the 2 prototypes

*P*-value < 0.05 was considered statistically significant

Visualization, Understanding score, Intended purchase score, and Ability to modify intended purchase: greater values represent better performance

## Discussion

Our study showed that a black-&-white stop sign with a simple text indicating ‘Excess of <nutrient>’ was the best option to implement a warning message in the context of the Chilean Food Labelling and Marketing Law. This label presented the highest visualization, understanding, and ability to modify intended purchase in Chilean low-middle SES women in charge of food purchases and adolescents. In June 2016, the Chilean government implemented this FoP warning label and currently, several countries such as Uruguay, Peru, and Israel have approved the implementation of warning labels similar to the one of Chile, while several other countries are in the process of political discussions (Brazil, Canada, India, among others) [16–20].

In contrast to most previous experiences with FoP labels that were based on positive messages, we only tested warning labels. There is sufficient evidence showing that excessive consumption of energy and some critical nutrients (such as sugars and sodium) increases the risk of obesity and obesity-related conditions such as hypertension, diabetes, etc. [40]. Therefore, dietary guidelines should consider decreasing the consumption of foods high in energy and critical nutrients and accordingly, FoP labelling should be designed to inform the population on which are the foods that contain higher energy and critical nutrients levels.

The warning label selected in this study had a very simple design; it was a black & white octagon as in a stop-sign and it only included the wording “Excess in” (see Additional file 2). Moreover, in the decree of implementation of the law precise indications were included regarding the size and location of the labels based on the area of the main display panel of the packaged foods. The selection of a simple label for providing the warning message is supported by our psycho-neurocognitive background. It might be intuitive that having more information will lead to better selections. This is actually the case for important and infrequent decisions (e.g. buying an apartment), when people usually look for detailed information in order to make a wiser decision. However, for frequent (i.e. daily) and relatively less important decisions, people usually use a shortcut in the decision-making process, minimizing the time and effort invested on those decisions [41]. Such strategies are known as heuristic tactics, and -based on our natural inclination to them- simple and directive nutrition labels (i.e. which need little or no interpretation) would be more effective in modulating food choices and diet. In fact, in our study the lay audience group meetings indicated a preference for detailed information in the labels; however, the results of the *Quantitative Phase* showed that simpler prototypes performed better than the ones displaying more information. In line with this evidence, recent experimental studies show a better performance of the Chilean warning label regarding attention, use, understanding and impact on healthier food choice (compared to GDA or traffic-lights) [13–15, 42]. Moreover, another experimental study reported an orange stop sign stating ‘WARNING: high sugar content’ having the greater impact on decreasing product preferences and likelihood to buy, compared to control condition, text warning, or price increase; similar results were only observed with plain packaging (a package without any marketing strategy) [42]. On the other hand, it has been argued that warning labels could be perceived as too harsh by consumers or reduce consumers’ control over food choices, which has been recently contradicted by a study performed among Canadian participants [43].

Several real experiences using FoP labels in packaged foods have been undertaken since this work was done. Ecuador implemented mandatory traffic-lights in 2014 (although they are not necessarily on the front face of the package) [44], Mexico implemented a mandatory GDA system in 2015 and Australia & New Zealand implemented the voluntary Health Star Rating in 2014. None of the already implemented FoP is a warning label, which makes Chile the first country to implement such mandatory warning FoP labels on pre-packaged foods. Countries such as Uruguay, Brazil, Peru, Canada, and Israel are currently in different stages of implementing similar mandatory FoP warning labels, and several other countries are starting the discussion on which FoP labelling system to consider. Results reported in this article might be of help for informing the discussion in different countries looking at strategies to tackle the obesity and NCD epidemics.

The present study has some limitations, as the lack of an experimental approach (contrasting with a control food package) in a more realistic setting (i.e. simulated purchasing or the use of real food packages) and the fact that we could not test all the relevant combinations of color, text or other important characteristics. However, the interdisciplinary and stepwise mixed methods approach (qualitative and quantitative) has allowed us to arrive to a prototype that seems to be working under real-setting conditions.

The Chilean MoH implemented the FoP warning label proposed in this article only replacing the words ‘Excess of’ by ‘High in’ because the Law wording did not allow the use of ‘Excess’. In order to make sure that people understood correctly the meaning of ‘High in’, a communication campaign was launched for presenting the new warning message [45] and the use of the word ‘High in’ as a positive attribute of foods (i.e. High in Vitamins) was banned [6]. Surveys conducted by the MoH at 6 and 18 months after implementation indicate that over 90% of consumers understand FoP warning labels and between 40 and 50% use them for informing their purchase decision (self-reported) [45, 46].

## Conclusions

In conclusion, after conducting a mixed-method stepwise study, a black-&-white stop sign warning label with a simple message: ‘High in <nutrient>’ was selected as the FoP warning label to be implemented in the Chilean Law of Food Labeling and Marketing. Several countries worldwide are discussing the implementation of similar FoP warning options. Current ongoing studies will be able to provide more information of the impact of this policy in a real-world setting.

## Additional files

Additional file 1: Prototypes tested on the Expert Group Meeting, description of the warning messages used on the prototypes tested in the expert group meeting. (DOCX 358 kb)

Additional file 2: Made-up yogurt used for displaying the different prototypes tested in the Quantitative Phase, graphic description of the made-up yogurt used for displaying the different prototypes tested. (DOCX 283 kb)

Additional file 3: Survey applied in the Quantitative Phase, Sub-studies #1 and #2. (DOCX 18 kb)

Additional file 4: Alternatives when more than one nutrient is excessive tested in the Quantitative Phase, Sub-study #2, description of the warning messages used on the prototypes tested in the quantitative phase, sub-study #2, when more than one nutrient was excessive. (DOCX 42 kb)

## Data Availability

The datasets used and/or analyzed during the current study are available from the corresponding author on reasonable request.

## Abbreviations

FoP: Front-of package
GDA: Guideline Daily Amounts
INTA: Institute of Nutrition and Food Technology
MoH: Ministry of Health
NCDs: Non-communicable diseases
SES: Socioeconomic status
