# Supplementary material for: Development of the Chilean front-of-package food warning label
Source: BMC Public Health. 2019 Jul 8;19:906. doi: 10.1186/s12889-019-7118-1 (PMC6615240; doi:10.1186/s12889-019-7118-1)
Supplement: Supplementary file 1 — Prototypes tested on the Expert Group Meeting, description of the warning messages used on the prototypes tested in the expert group meeting. (DOCX 358 kb) [file 12889_2019_7118_MOESM1_ESM.docx]

**Additional File 1. Prototypes tested on the Expert Group Meeting.**


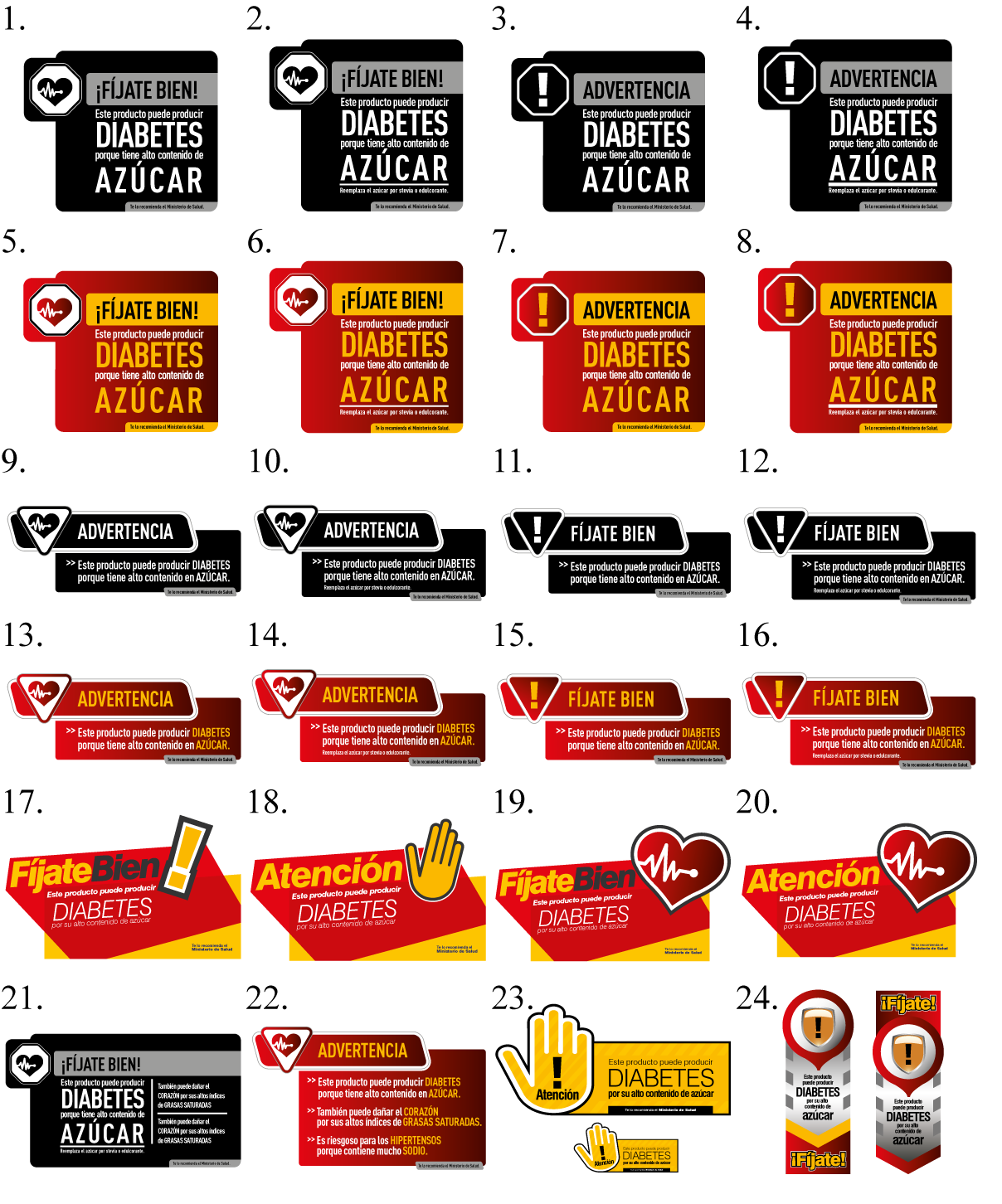


Text of prototypes 1, 5, 11, 15, 17, 19 & 24 reads: *¡Fíjate bien! Este producto puede producir diabetes porque tiene alto contenido de azúcar. Te lo recomienda el Ministerio de Salud*= Pay attention! This product may cause diabetes given its high content of sugar. This is a Ministry of health’s recommendation;

Text of prototypes 2, 6, 12 & 16 reads: *¡Fíjate bien! Este producto puede producir diabetes porque tiene alto contenido de azúcar. Reemplaza el azúcar por stevia o edulcorante. Te lo recomienda el Ministerio de Salud*= Pay attention! This product may cause diabetes given its high content of sugar. You should replace sugar by stevia or another non-caloric sweetener. This is a Ministry of health’s recommendation;

Text of prototypes 3, 7, 9 & 13 reads: *Advertencia. Este producto puede producir diabetes porque tiene alto contenido de azúcar. Te lo recomienda el Ministerio de Salud*= Warning! This product may cause diabetes given its high content of sugar. This is a Ministry of health’s recommendation;

Text of prototypes 4, 8, 10 & 14 reads: *Advertencia. Este producto puede producir diabetes porque tiene alto contenido de azúcar. Reemplaza el azúcar por stevia o edulcorante. Te lo recomienda el Ministerio de Salud*= Warning! This product may cause diabetes given its high content of sugar. You should replace sugar by stevia or another non-caloric sweetener. This is a Ministry of health’s recommendation;

Text of prototypes 18, 20 & 23 reads: *Atención. Este producto puede producir diabetes porque tiene alto contenido de azúcar. Te lo recomienda el Ministerio de Salud*= Attention. This product may cause diabetes given its high content of sugar. This is a Ministry of health’s recommendation;

Text of prototype 21 reads: *¡Fíjate bien! Este producto puede producir diabetes porque tiene alto contenido de azúcar. Reemplaza el azúcar por stevia o edulcorante. También puede dañar el corazón por sus altos índices de grasas saturadas. Te lo recomienda el Ministerio de Salud*= Pay attention! This product may cause diabetes given its high content of sugar. You should replace sugar by stevia or another non-caloric sweetener. It also can damage your heart because of its high content of saturated fats. This is a Ministry of health’s recommendation;

Text of prototype 22 reads: *Advertencia. Este producto puede producer diabetes porque tiene alto contenido de azúcar. También puede dañar el corazón por sus altos índices de grasas saturadas. Es riesgoso para los hipertensos porque tiene mucho sodio*= Warning. This product may cause diabetes given its high content of sugar. It also can damage your heart because of its high content of saturated fats. It is a risk for hypertensive people given its high content of sodium. This is a Ministry of health’s recommendation.
