# Supplementary material for: Development of the Chilean front-of-package food warning label
Source: BMC Public Health. 2019 Jul 8;19:906. doi: 10.1186/s12889-019-7118-1 (PMC6615240; doi:10.1186/s12889-019-7118-1)

**Additional File 2. Made-up yogurt used for displaying the different prototypes tested in the Quantitative Phase.**


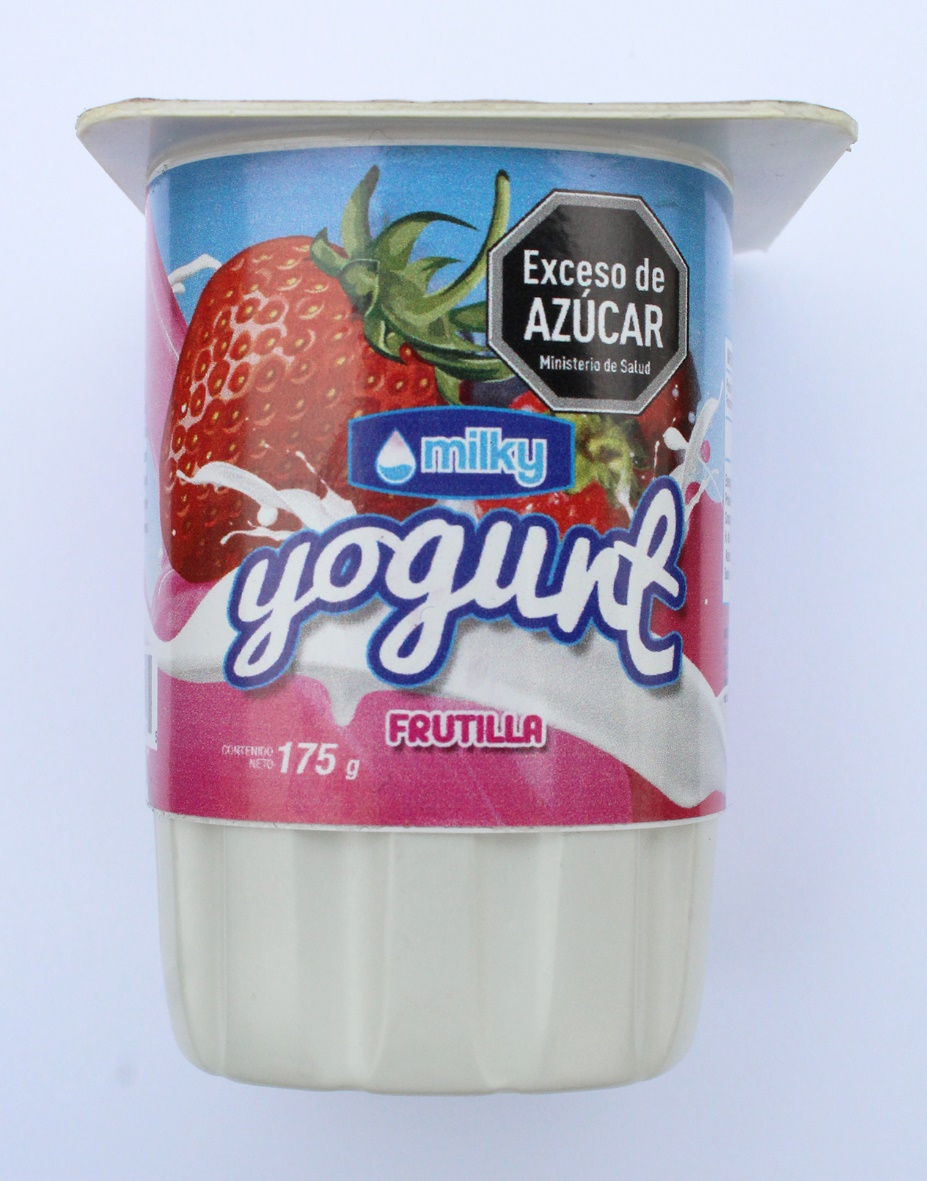

Supplement: Supplementary file 2 — Made-up yogurt used for displaying the different prototypes tested in the Quantitative Phase, graphic description of the made-up yogurt used for displaying the different prototypes tested. (DOCX 283 kb) [file 12889_2019_7118_MOESM2_ESM.docx]
