# Supplementary material for: Development of the Chilean front-of-package food warning label
Source: BMC Public Health. 2019 Jul 8;19:906. doi: 10.1186/s12889-019-7118-1 (PMC6615240; doi:10.1186/s12889-019-7118-1)
Supplement: Supplementary file 3 — Survey applied in the Quantitative Phase, Sub-studies #1 and #2. (DOCX 18 kb) [file 12889_2019_7118_MOESM3_ESM.docx]

**Additional File 3. Survey applied in the Quantitative Phase, Sub-studies #1 and #2.**

| 0. Sex* | 1. Male  2. Female |
| --- | --- |
| 1. Age*** | _______ years  Alternatively, an age range can be selected  1. <18y 🡪 Survey ends  2. 18-24y  3. 25-30y  4. 31-39y  5. 40-49y  6. 50-59y  7. ≥60y 🡪 Survey ends |
| 2. Neighborhood | __________________  If interviewee does not live in the neighborhood 🡪 Survey end |
| 3. Are you one of the main responsible of food purchases? ** | 1. Yes  2. No 🡪 Survey ends |
| 4. Do you usually buy yogurts? *** | 1. Yes  2. No 🡪 Survey ends |
| 5. How many units of yogurt do you buy per week? *** | _______ units  If <4 units/week 🡪 Survey ends |
| 6. How healthy you think yogurts are? | 1. Very healthy  2. Healthy  3. Indifferent  4. Unhealthy  5. Very unhealthy |
| 7a. For how many people you buy yogurts? ** | _________ people |
| 7b. How many of them are under 15y? ** | _________ people |
| 8. Are you studying currently? ** | 1. Yes  2. No |
| 8 **  If question 8=Yes🡪 What’s your current educative level?  If question 8=No🡪 What’s your last approved level? | 1. Never assisted  2. Special education (differential)  3. Primary of preparatory (old system)  4. Primary school  5. Humanity or Commercial technique, Industrial or Normalist (old system)  6. Secondary school  7. Superior level technic  8. Professional  9. Master or PhD  99. Do not know |
| 9. Is there anything on the label of this product that attracts your attention? (three more important) | 1. ____________________________  2. ____________________________  3. ____________________________ |
| Check 1. The interviewed identified the warning label? | 1. Yes  2. No 🡪 interviewer must show the warning label and explain that following questions are focused on the label |
| 10. How notorious is this message? | 1. Not notorious at all  2. Not notorious  3. Indifferent  4. Notorious  5. Very notorious |
| 11. How easy to understand is this message? | 1. Very difficult to understand  2. Difficult to understand  3. Indifferent  4. Easy to understand  5. Very easy to understand |
| 12. According to this message, the excessive consumption of this product is (…) | 1. Very healthy  2. Healthy  3. Indifferent  4. Unhealthy  5. Very unhealthy |
| 13. According to this message ¿which of the following nutrients is excessive? | 1. Calories  2. Sodium  3. Calcium  4. Saturated fats  5. Sugar |
| 14. According to this message, you should consume (…) | 1. A lot more of this product  2. A bit more of this product  3. Same as usual  4. A bit less of this product  5. Nothing at all of this product |
| 15. How trustful is the information of this message? | 1. Not trustful  2. Less trustful  3. Trustful  4. Very trustful  5. Completely trustful |
| 16. If the yogurt you usually buy had this message, would you buy it? *** | 1. I would buy it for sure  2. It is likely that I would buy it  3. Indifferent  4. It is unlikely that I would buy it  5. I would not buy it |
| 17. If the yogurt you usually buy had this message, how many products would you buy per week? *** | __________ units |
| 18. If the yogurt you usually buy had this message, would you prefer to buy another one without the message, but with similar price, flavor and brand? *** | 1. I would buy it for sure  2. It is likely that I would buy it  3. Indifferent  4. It is unlikely that I would buy it  5. I would not buy it |
| 19. If the yogurt you usually buy had this message, would you prefer to buy another one without the message, even if more expensive? *** | 1. I would buy it for sure  2. It is likely that I would buy it  3. Indifferent  4. It is unlikely that I would buy it  5. I would not buy it |
| 20. If the yogurt you usually buy had this message, would you prefer to buy another one without the message, even if less tasty? *** | 1. I would buy it for sure  2. It is likely that I would buy it  3. Indifferent  4. It is unlikely that I would buy it  5. I would not buy it |
| 21. If the yogurt you usually buy had this message, would you prefer to buy another one without the message, even from an unknown brand? *** | 1. I would buy it for sure  2. It is likely that I would buy it  3. Indifferent  4. It is unlikely that I would buy it  5. I would not buy it |
| 22. When you buy foods, how important are the nutritional characteristics of the product in your purchase decision? | 1. Not important at all  2. Not very important  3. Indifferent  4. Important  5. Very important |
| 23. When you buy packaged foods, how often you read the nutrition fact panel? | 1. Never  2. Almost never  3. Sometimes  4. Frequently  5. Always |
| 24. Which is your height? | _________ meters  Alternatively, a height range can be selected  1. < 1.40 m  2. 1.41 - 1.50 m  3. 1.51 - 1.60 m  4. > 1.61 m |
| 25. Which is your weight? | _________ kilograms  Alternatively, a weight range can be selected  1. < 45 kg  2. 46 - 60 kg  3. 61 - 70 kg  4. > 70 kg |
| 26. Has any health professional told you -or a family member- have/has diabetes? | 1. Yes  2. No |
| *The following three questions were done only in Sub-study #2, with the interviewer showing 2 prototypes (in the made-up yogurts), regardless of which of them was evaluated* | |
| 27a. Based on the warning labels, rank these 2 yogurts from the one with a more notorious label to the one with a less notorious label | 1. First place: _________  2. Second place: _________ |
| 27b. Based on the warning labels, rank these 2 yogurts from the one with easier-to-understand-label to the one with the harder-to-understand-label | 1. First place: _________  2. Second place: _________ |
| 27c. Based on the warning labels, rank these 2 yogurts from the one with the greater ability to reduce your purchase to the one with the lower ability | 1. First place: _________  2. Second place: _________ |
| *The following three questions were done only in Sub-study #2, with the interviewer showing 2 alternatives for displaying more than one nutrient in excess (Supplemental Figure 3)* | |
| 28a. In the case that more than one nutrient is excessive, which of these 2 ways of presenting the message is easier for you to see? | 1. Option 1  2. Option 2 |
| 28b. In the case that more than one nutrient is excessive, which of these 2 ways of presenting the message is easier for you to understand? | 1. Option 1  2. Option 2 |
| 28c. In the case that more than one nutrient is excessive, which of these 2 ways of presenting the message would have greater influence in your purchase decision? | 1. Option 1  2. Option 2 |

* Question was only asked to adolescents, not applied to women.

** Question was only asked to women, not applied to adolescents.

*** Version applied to women, a different version was applied to adolescents. For Q1, age ranges were different for adolescents (1. <12y, 2. 12-14y, 3. 15-17y, 4. 18-24y, 5. 25-30y, 6. >30y). For Q4, Q16, Q17, Q18, Q19, Q20 and Q21, adolescents were asked about yogurts consumption instead of yogurts purchase. For Q5, adolescents were asked about monthly consumption instead of weekly purchase.
