# Supplementary material for: Development of the Chilean front-of-package food warning label
Source: BMC Public Health. 2019 Jul 8;19:906. doi: 10.1186/s12889-019-7118-1 (PMC6615240; doi:10.1186/s12889-019-7118-1)
Supplement: Supplementary file 4 — Alternatives when more than one nutrient is excessive tested in the Quantitative Phase, Sub-study #2, description of the warning messages used on the prototypes tested in the quantitative phase, sub-study #2, when more than one nutrient was excessive. (DOCX 42 kb) [file 12889_2019_7118_MOESM4_ESM.docx]

**Additional File 4. Alternatives when more than one nutrient is excessive tested in the Quantitative Phase, Sub-study #2.**


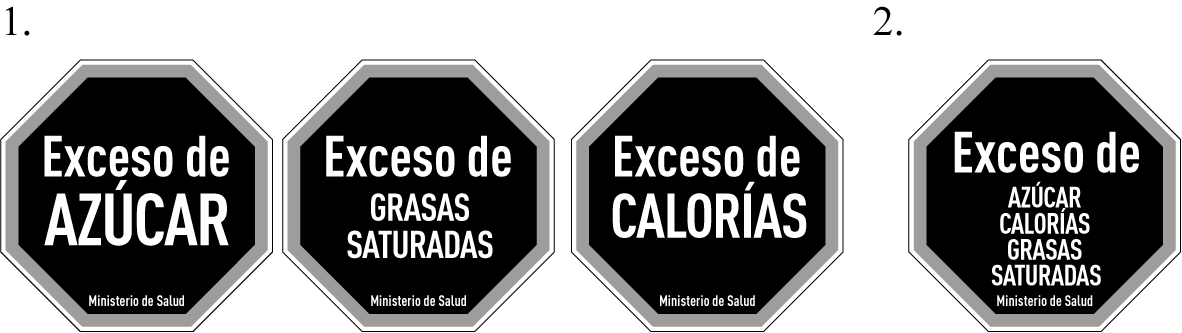


1. One stop sign per critical nutrient in excess. *Exceso de azúcar*= Excess of sugar; *Exceso de grasas saturadas*= Excess of saturated fats; *Exceso de calorías*= Excess of calories.

2. One stop sign for all critical nutrients in excess. *Exceso de azúcar, calorías, grasas saturadas*= Excess of sugar, calories, saturated fats.
